# If times change, should we throw away the hearthstone? Exploring (Dis) continuities in autonomy and decision-making in the lives of Ghanaian women

**DOI:** 10.3389/fpsyg.2014.01371

**Published:** 2014-11-28

**Authors:** Vivian A. A. Dzokoto, Akosua K. Darkwah

**Affiliations:** ^1^African American Studies, Virginia Commonwealth UniversityRichmond, VA, USA; ^2^Department of Sociology, Centre for Gender Studies and Advocacy, University of GhanaLegon, Ghana

**Keywords:** Ghana, gender, women, West Africa, proverbs

## Abstract

This paper attempts to investigate continuities and discontinuities between traditional and modern representations of womanhood and female gender roles focusing primarily on family and work settings. Using approaches informed by Sociology, Cultural Psychology, and African Studies, the paper explores traditional views of womanhood encapsulated in (and also transmitted intergenerationally) through proverbs. This customary perspective is contrasted with the results of the Everyday Lives Survey from the Pathways of Women's Empowerment Ghana project. The survey investigated the nature of everyday life– education, work, decision making, access to institutions, and autonomy in relationships—in six hundred (600) adult women in both rural and urban communities in three regions of Ghana. We argue that although the times are changing, there have only been modest disruptions in the lives of Ghanaian women as far as issues of autonomy and decision-making in are concerned.

## Introduction

Mmere dane a, ye dane ye ho bi

If times change, we change ourselves too (Bu Me Be, #343)

The West African Nation of Ghana, formerly known as the Gold Coast, has undergone significant change in the 57 years since its independence from British rule. Its population has grown from 6.7 to 25 million, and it has experienced a mixture of democratic and military rule, as well as economic failures and some growth. The state of the Ghanaian family in general, and the experience of being female in particular have undergone change during this period as well. For instance, Takyi (2003) observed a decline in parental involvement in spousal selection. Oheneba-Sakyi and Takyi (2006) posited that modernization (in the form of increased education, large rural-urban migration and growth within urban spaces) has impacted African families in three major ways: (i) Decreased length of post-partum sexual abstinence (traditionally used as a means of child spacing); (ii) A shift from exclusive breast feeding to varying degrees of weaning foods and supplements; and (iii) An increase in marital instability, resulting in an increase in women-headed households. In addition, Ardayfio-Schandorf (2006) noted changes in (i) the legal definition of marriage, (ii) in the social and legal status of women and children, and (iii) an increase in female-headed households, attributable to economically –motivated migration by their partners, and weakened extended family ties. Finally, analysis of three waves of the Ghana Demographic and Health Survey by Heaton and Darkwah (2011) found that over time, Ghanaian women were waiting longer to commence sexual relationships, wed, and become mothers. Ghana has also witnessed the fastest decline in total fertility rates in the West African sub-region going from 6.4 to 4.0 over a 15 year period (Ghana Statistical Service, 2008).

Today's Ghanaian woman is by no means a complete reinvention; many patterns of traditional values and norms have been retained despite the decades of social change. For instance, analysis of the Ghana Demographic and Health Survey by Heaton and Darkwah (2011) found only modest declines in frequencies of marital disruption, polygamy, and informal unions over time. Ardayfio-Schandorf (2006) observed that “several aspects of the traditional family can still be identified alongside other emerging family norms” (p. 129). Thus, the role of the Ghanaian woman within the modern Ghanaian family—which serves as the backbone to Ghanaian society—is a combination of the new and the old.

### Psychological approaches

Understanding what has changed and what has not is important to accurately determine the challenges and opportunities in the lives of Ghanaian women today. Research about the Ghanaian family and females has primarily been conducted from socio-demographic perspectives. Such approaches, while important, are not exhaustive. Exploring psychological perspectives are important to fully understand the needs and realities of contemporary families. Multiple approaches in psychology are relevant to the study of Ghanaian families. For example, the cultural psychology perspective can be used to explore the mutual and dynamic constitution of interpersonal relationship tendencies and cultural-ecological drivers of abstracted independence and neoliberal subjectivity (Salter and Adams, 2012). Additionally, the Family Psychology approach—based on the premise that family dynamics are central to the psychological functioning of family members (Bray and Stanton, 2009)—is a general systems theory-based approach useful for exploration of psychological dimensions of the lives of women in the context of the nuclear and extended family networks in which they are embedded.

The goal of our paper is to identify areas of intergenerational change in the lives of Ghanaian women that have relevance for psychological indicators such as determinants of self-worth and sources of distress. Our data exploring social change in women comes from two distinct sources. Proverbs served as a source of information about “the traditional Ghanaian woman.” Contemporary information was obtained from the results of the Everyday Lives Survey from the Pathways of Women's Empowerment Ghana project.

Our decision to use proverbs as a source of information about traditional values relating to womanhood was influenced by Markus and Kitayama's (1994; 2010) situation of cultural influences on the self partly within cultural products and culturally pervasive ideas. Lamoreaux and Morling (2012) observe that cultural products lend themselves well to analysis of cultural dynamics and allow for investigations of cultural influences without the risk of a variety of biases associated with self-report. Members of society learn about, internalize and incorporate societal ideas in their thoughts, feeling and behavior, and by doing so, create patterns, actions, products and discourse that reproduce, and on occasion, modify the prevailing sociocultural narrative. In Ghanaian and other African societies, proverbs—succinct statements that carry a message—are ubiquitous cultural products that collectively encapsulate cultural norms and standards. Due to their prominence in African societies, proverbs have been an important focus of investigative inquiry in the field of African Studies. African proverbs have been described as value repositories (Yankah, 2000; Hussein, 2005). They actively serve as socialization tools used to advise, reprimand, instruct, encourage, settle conflicts, and remind people of societal values (Gyekye, 1996; Yankah, 2000). In other words, in African society, proverbs are cultural products that store, and communicate, cultural ideas about what is good, just, and moral, and transmit them intergenerationally. Mbiti (1997), in an editorial of an African proverb series summarized the importance of proverbs as follows: “in short, proverbs present an opportunity for interdisciplinary exploration and discussion in various fields, including religion and philosophy, sociology and anthropology, linguistics and literature, history, geography, economics, medicine and communication.” Hussein (2009) observed that an important impact of proverbs in African societies has been the reflection, maintenance and reconstruction of a gendered social world.

The extent to which African proverbs reflect traditional African values has been addressed by several African Studies scholars (see Mbiti, 1975, 1993; Opoku, 1975; Yankah, 1986; Gyekye, 1996). Opoku (1975) stated that African proverbs “express the wisdom of the African people and are a key to the understanding of African ways of life in the past” (p. 8). Twenty two years later, Opoku revisited this question and maintained that “proverbs are encapsulations of the accumulated wisdom and experiences of past generations of the Akan and they constitute an authentic mirror of the mind and philosophy of the Akan people” (Opoku, 1997, p. xviii). In his seminal book *African Cultural Values: An Introduction*, Gyekye, a renowned Africanist Philosopher, (1996) demonstrates that African cultural values “can be extracted from … beliefs, practices, institutions, myths, folktales and proverbs” (p. xiii). He demonstrates extensive connections between specific proverbs [such as “No one shows the Supreme Being to a child (p. 7); Humanity has no boundary (p. 27), Man's brother is man (p. 28)”] and identified cultural values and beliefs such as major tenets of African Traditional religion; notions of humanity and brotherhood; importance of ancestors; and moral, economic, family, political, and aesthetic values. Similarly, Brookman-Amissah (1986) points out the parallels between Akan proverbs about death (in five distinct domains: Nature and Universality of Death, Causes of Death, Inheritance, Death, and the Hereafter, God and Death) and traditional Akan funeral practices. Yankah (1986) demonstrates connections between Akan proverbs addressing morality and behavior in specific interpersonal contexts and the rulings of traditional (as distinct from Western-style) Akan courts. Yankah's work is consistent with Christensen's (1958) observation that particular Akan proverbs are regarded as the “verbalization of customary law” (p. 242). In the same work which reviewed the roles of proverbs in an Akan subgroup, Christensen commented that proverbs “may prove an aid to the ethnographer in ascertaining the ideal norms of behavior” (p. 242) within traditional society. In his book *Tradition and Modernity: Philosophical Reflections on the African Experience*, Gyekye (1997) points out that “SubSaharan Africa has received” “ideas, practices and institutions… from other cultural sources” (p. 26), and concludes that post-colonial African culture has influences both from traditional African and Western cultural forces. Given that proverbs are cultural products produced by indigenous rather than external influences, they are more consistent with traditional values than post-colonial ones. Gyekye (1997) nevertheless considers proverbs to be relevant to contemporary Africa “when the appropriate and necessary amendments and refinements have been made” (p. 171). The precise degree of concurrence of contemporary African values and lifestyle with traditional values espoused by proverbs is unclear and warrants investigation.

One of the largest published collections of African proverbs is from the Akan, who constitute Ghana's largest ethnic group. Cultural similarities within Ghanaian ethnic groups suggest that many of the culturally disseminated and shaped values about women communicated by proverbs are not uniquely Akan. Marriage, for example, is a societal expectation across the 10 geopolitical regions of the country regardless of ethnicity. Similarly, the importance of childbearing is shared as strongly among the Akan population as it is among the non-Akan population. Other aspects of Ghanaian culture that used to be unique to the Akan have been embraced by the other ethnic groups in the country. For instance, the complementary political system where women represented by the Queenmother have their spheres of influence and men represented by the Chief have their own (Manuh, 1988) has been adopted by the other ethnic groups in the country although the powers that Queenmothers have in other Ghanaian societies is not at par with that of Queenmothers in Akan societies. We thus infer that values captured in proverbs are cultural products that do not simply act as social representations (Moscovici, 1972) of the Akan, but of traditional Ghanaian culture. This approach is consistent with Gyekye's work (Gyekye, 1996), which justifies the extrapolation of values beyond the Akan social sphere based on “sufficient commonalities in many areas” (p. xiv) of African culture.

Information about the status of women in contemporary Ghana can be gleaned from a plethora of sources, using a variety of methodological approaches. Our decision to use survey data was shaped by the demonstrated advantages of this research method including efficiency, generalizability, and versatility (Fowler, 2014). While several waves of the Ghana national census data are available, the items covered therein do not allow for an in-depth examination of the day-to-day experiences in the lives of Ghanaian women, especially in the area of empowerment. This is largely because demographic data typically tends to focus simply on the outcomes of processes that may have relevance for the empowerment of women, rather than on the empowerment dynamic of such processes or interpersonal interactions. For instance, while national surveys do provide information about number of children living in a household, they do not examine related sexuality issues such as the extent to which sex is viewed as a source of pleasure vs. simply as a procreational process (Pereira, 2003). Feminist scholars such as McFadden (2003) have (i) argued that sexuality needs to be conceptually distinguished from reproduction, (ii) demonstrated the relationship between power and sexual pleasure, and (iii) implied that the importance ascribed to female sexual pleasure, even in the context of legally-sanctioned long-term, heterosexual, committed relationships, is an index of feminist choice. Furthermore, the national census does not explore the adherence with traditional values such as those captured by proverbs. As such, detailed information about the state of affairs in the lives of contemporary Ghanaian women is best gleaned from surveys designed to explore decision-making and empowerment, such as the Ghana Everyday Lives Survey.

## Methods

### Data

Our proverbs source was Appiah et al. (2007) published compendium of 7015 Akan proverbs. The compendium presents each proverb in the original Akan, with an English literal translation of each proverb, accompanied by the English meaning of the proverb. For example, The Akan proverb *Mmere dane a, yedane yen ho bi* (#343) translates literally as “If times change, we change ourselves too.” The manifest meaning of the proverb provided by the authors is as follows: “You must adapt yourself to circumstances as they are” (p. 29). On occasion, the compendium provided additional context (e.g., an explanation of a word, summary of a historical event, reference to a story) to help the reader understand the proverb. For example, The Akan proverb O*baa bone na ycfiri no ntoma kokoo* translates literally as “A bad woman, we give her a red cloth on credit.” The authors provide the following context to explain the proverb: A red cloth is used for funerals. Then the manifest meaning is provided: We want trouble to come to those we dislike (p. 14). On occasion, the literal translation and manifest meaning of the proverb were the same in which case only one meaning was provided in the compendium.

The ethnographic technique of cultural domain analysis, which considers text as an indicator of human experience (Tesch, 1990) was used to guide the thematic analysis of the proverbs, which was conducted by a bilingual (Akan, English) co-author and an English-speaking research assistant. Each proverb was characterized as a unit of analysis that could have one or more associated themes. After an initial review of all 7015 proverbs, a subset of female-related themes were manually extracted from the larger collection by the research assistant, using a filter of female-related keywords such as woman, child, work, lady, girl, birth, mother, marry, family, maternal, and derivatives thereof (e.g., womanhood, mother^*^, etc.) for both literal and manifest proverb translations. Next, the bilingual researcher identified proverbs that were consistent with the foci of the Everyday Lives Survey. An intra-rater check on the reliability of the categorizations was conducted by re-categorizing randomly selected data from the original, final and intermediate subsets of proverbs at different times.

### Survey and participant overview

The Everyday Lives Survey was administered in 2008 to 600 women randomly selected from three age groups: 18–29; 30–49; above 50. Since Ghana's population is relatively young, the 18–29 year olds constituted 40.3% of the sample, the 30–49 year olds constituted 39.5% of the sample while those aged above 50 constituted 20.2%. The women who participated were from both rural and urban communities in three regions of Ghana (reflecting the ecological and ethnic diversity of the country). As part of a larger project of which the second author was a part, this survey was conducted to explore the ways in which Ghanaian women's lives had changed for better or worse since Ghanaian independence in 1957. Graduate students with experience in administering surveys and bilingual in English as well as one other Ghanaian language administered the surveys in the appropriate Ghanaian language. The survey investigated the nature of everyday life– education, work, decision making, access to institutions, and autonomy in relationships. For purposes of this article, we looked specifically at the following seven variables: ability to keep a baby even if partner does not want it; having a say in whom one married; belief in sex for pleasure; contraceptive usage; the decision to build a house, the decision to purchase a large item; and the decision to buy a productive (investment) asset. Four of the questions had a yes/no response format (e.g., Did you have a say in who you entered into a marriage with the first time?), while three items had a multiple categorical response format (e.g., In your household, who makes the decision to buy a large item?) (See Supplementary Material).

The 600 women recruited from urban and rural areas in Northern, Central, and Southern Ghana generally had low levels of education. Only a little more than half the sample (55.5%) had been to school. Slightly more than a third (37.9%) reported having completed basic education while 13.0% had completed Secondary School. Less than five percent of the respondents (4.6%) had tertiary education. Educational levels of women have improved over time and it is evident in the fact that many more of those aged 18–29 (76.5%) have had the privilege of formal education as compared to 31.6% of those aged above 50. Educational rates are also skewed by geographic location with more females in urban areas likely to have access to post-primary education (Amu, 2005).

Marriage was also fairly universal for this group. Only 16%, the majority aged between 18 and 29, have never been married. A quarter of the sample (23.2%) is in consensual unions and 39.5 percent are married. Ten percent are widowed, 4.8% are divorced and 6.5% are separated. The socio-demographic characteristics of the sample discussed here are fairly similar to that of the national census undertaken in 2010. This is important to note because it suggests that patterns observed in our sample may be taken as an index of what occurs in the wider Ghanaian society.

These two data sources—the proverbs and the survey—provide us with complementary views about the lives of Ghanaian women. On the one hand, the proverbs illuminate what traditional Ghanaian society considered normal (If a woman buys a sheep, it is a man who rears it; # 95), aspirational (A woman who can produce 10 children eats the tenth child's sheep; # 6675), and taboo (A woman sells garden eggs, but she doesn't sell gunpowder; # 96) manifestations of womanhood and femininity. Such values have survived transmission across generations. In contrast, the Everyday Lives Survey provides a cross-sectional glimpse into the lives of women in contemporary Ghana. Taken together therefore, these two data sources provide information about both long term and short term social change. Admittedly, a short coming of the proverb-inferred information is the unavailability of information on the extent to which these values were attained. Hence, while we can be certain that those values were at least aspirational in nature, there is no evidence permitting us to estimate the extent to which such values were in fact attained within traditional settings in Ghana's past. Additionally, proverbs are historically imprecise cultural artifacts since it is impossible to situate them in an exact period in history. Nevertheless, the proverbs provide us with a rough baseline from which to begin to estimate and understand the potential reach of social change. Due to the inherent limitations of cross-sectional research designs, we relate the survey findings where possible to other relevant research on Ghanaian women.

## Results and discussion

### Social changes in the autonomy of ghanaian women

*If the hardworking/ideal woman has a child on her back, she also carries a load at the same time (#77)*.

The proverb above addresses the multiplicity of roles that traditional society expected women to play. It projects the imagery of a woman with a child on her back. The Ghanaian cultural practice of putting children on a mother's back differs from the western piggy-back ride in a marked way: the child is securely attached to the mother by two sets of cloth that the mother wraps around and then ties around her chest. This traditional way of carrying young children in Ghana frees up the mother's arms. The first part of the proverb communicates the traditional expectation of reproductive responsibilities—the traditional woman's role of mother and the associated duties of childcare. The notion of work is symbolized by the imagery of the woman carrying a physical load, (which is usually done by balancing the load on one's head). This part of the proverb refers to the (economic) productive activities of a woman which in traditional Ghanaian society inevitably involves a woman carrying a load on her head (e.g., farming implements or produce (in the case of a farmer), wares for sale (in the case of a small-scale retailer), or food for sale (in the case of a baker, for example). Thus, the proverb implies that the traditional trope of ideal womanhood involves both reproductive and economically productive roles; in other words, the ideal traditional woman is the working mother.

Work in the traditional Ghanaian setting was as crucial for women as it was for men and was crucial for purely instrumentalist purposes. Women needed to work to be able to fulfill their obligations to both their families of origin (parents, siblings) and their families of reproduction (children especially). Women were expected to be able to contribute, for example, to funeral expenses of matrilineal/patrilineal cousins as well as provide daily sustenance for children. In the past, parents fulfilled the dietary needs of children in the following manner: mothers provided the carbohydrates and fathers the protein needs (Clark, 1994). Over time, this has been transformed into a situation where partners have separate finances or what Ekejuiba (1995) refers to as separate purses and distinct financial responsibilities: mothers provide the basic needs of a family (food, clothing) while fathers cover the more expensive items such as housing needs and school fees. Ability to work so as to be able to take care of the financial needs of children is seen as more important than the need to respond to the physical needs of a child. As one mother explained, “Everyone likes children, so they would not let them stay hungry or hurt themselves, but no one would work for them the way I do” (Clark, 1994, p. 360). Being a stay-at-home mother therefore does not make sense in the Ghanaian cultural context. Women are expected to be visible and active in both the public and private sphere. Labor force participation rates of Ghanaian women are therefore quite high. In fact, Tzannatos (1999) has argued that for every 100 Ghanaian men working, there are 101 Ghanaian women working.

While there were no proverbs that explicitly stated that women should work, the concept of a working woman was captured by proverbs that explored gendered work, and referred to women selling and engaging in farm work. The ideal of a hard working woman was captured by several proverbs such as *“If a hard-working woman gets married, she brings good things into the house”*# 44. In contrast, laziness was discouraged by proverbs such as *“If a lazy woman goes to get married, they take a broom to drive her away*” #62.

Women's economic independence is fairly deeply entrenched in Ghanaian society; only 0.2 percent of the women sampled did not think that it was crucial for a woman to have her own source of income. This is becoming even more important in contemporary Ghana where the incidence of Female Headed Households is on the rise, attributed primarily to rural-urban drift (Amu, 2005) and international migration (Development Research Centre, 2007) of male partners for economic reasons (Ardayfio-Schandorf, 2006).

The work that women can engage in is shaped in part by their educational attainment. We were unable to find proverbs that addressed formal education in women in the proverb compendium. Given the generally low levels of formal education at the secondary level and beyond, the majority of Ghanaian women work in jobs that do not require a great deal of academic preparation. Consequently, the majority of women (91.8%), according to the Ghana Living Standards Survey (GLSS) of 2008 work in Ghana's informal economy, centering primarily in agricultural (food and animal production and processing: 48%) and sales (wholesale and retail; 20%) jobs. In contrast, 8.2% of Ghanaian women above the age of 15 are employed in the formal economy (Ghana Statistical Service, 2008, p. 36).

While the proverbs reviewed indicate that the idea of a working woman appears consistent with traditional expectations of womanhood in Ghana, women's lives are inextricably linked with their spouses and extended families. Traditional values espoused that no matter how much money a woman made, she was generally not expected to have an independent existence.

Obaa wo mpempem a, Obarima na Ohwe ne Soo. (#98)

*English Translation: However rich a woman may be (lit. If a woman has thousands and thousands), it is a man that looks after her*.

*Meaning of Proverb: Whatever a woman may do she needs a man*.

Obaa osene boama/[ye kyem] a, etwere Obarima dan mu. (#97)

*English Translation: (Even) if a woman [carves a drum/[makes a shield], she keeps it in a man's room*.

*Meaning of Proverb: Whatever a woman may do she needs a man*.

Clearly, women were expected to have lives that were far from independent. They were expected to rely upon their extended family and/or their husbands. While the role of the extended family is acknowledged in some proverbs, a more prominent message captured by the corpus of proverbs reviewed is one of dependence on men (*If the soup will be good, it is because of a man* #3912), attribution of women's success to men (*If a woman does well (i.e., becomes rich), it is because of a man* #102), and the linking of women's socially desirable attributes to men (*If a woman is beautiful, it is because of a man* #54).

For these reasons, marriage was considered extremely important. This is illustrated by proverbs such as *#70. Obaa animuonyam ne awaree*.

*Translation and Meaning: A woman's glory (lit. what causes her to be respected) is marriage*.

The ramifications of the high value placed on marriage in this traditional cultural context include the connection of marital status to perceptions of worth of women and the maintenance of marriages once they occur (resulting in relatively low divorce rates, as observed in the survey sample). Traditionally, divorce resulted in a loss of social approval, as illustrated by the proverb: *Obaa biara te se deebenara, na Ogyae awaree, gyae awaree a, n'anim mma nyam. ' No matter how beautiful a woman is, if she is always divorcing (lit. stops marriage) she has no respect*.(#39). These sentiments are consistent with data from Ghana's last census as well as the Everyday Lives Survey, which reported low rates of divorce (less than 5% in both cases). In addition, single status was reported by 16% of the Everyday Lives sample, the majority of whom were aged under 30. Although the percentage of reported unmarried women was higher in the national sample (42%), it is important to take note that this statistic is based on a national representative sample of females aged 12 and above.

While it is clear that Ghanaian women of today (and yesterday) are expected to work and generate income, the data reviewed so far does not adequately speak to the state of Ghanaian women in terms of autonomy outside the financial sphere especially given the proverbs about dependence on men. In order to explore this issue further, we examine data on decision-making abilities.

### Decision making by Ghanaian women

*#80. Obaa pa ne dee Otie ne kunu asem*.

*A good woman is she who listens to her husband's advice*.

Given the non-autonomous state of women dictated by traditional norms, as illustrated by the proverb above, it stands to reason that traditionally, decision-making would occur in conjunction with others (in particular the spouse and extended family). Important life events affecting the lives of women that may involve decision-making include choice of marital partner, decisions concerning having children, and financial decisions impacting the household. The sentiments of the aforementioned proverb indicate that women would not be expected to make such decisions by themselves, but rather seek the input of others. The traditional practice of kin involvement in spousal selection is captured in proverbs such as “*If you give your child to a frog in marriage, she gives birth to a long-legged frog”* (#1359), which served as a cautionary message against making lackadaisical spousal pairings (the non-desirable spouse in this case being symbolized by the frog). While women were given the opportunity to assent to the marital arrangement in some cases, women were neither primarily nor exclusively responsible for choosing marriage partners.

The societal value placed on childbirth and having large numbers of children is represented by proverbs such as *Congratulations for giving birth can never end;* (#4199), and *A woman who can produce 10 children eats the tenth child's sheep*; (#6675). The latter proverb refers to the practice of women being presented with a sheep in honor of bearing a tenth child. Thus, it stands to reason that traditionally, women's decisions on whether to have children would have been strongly influenced by the advice of others consistent with the cultural norm.

How autonomous are Ghanaian women in decision making today? We measured decision making using three variables: choice in marital partner, choice to keep a baby and contributions to decision-making regarding purchases in a household. A majority of respondents (86.8%) had a say in the choice of marital partner. Although there was a 13.6% point difference between the percentage of women aged above 50 (79.3%) who were able to choose their marital partner and those aged 18–29 (92.9%) who could do same, this difference was not statistically significant (*p* = 0.934). We describe this change then as a non-significant change.

With respect to the extent to which male partners are in charge of decision-making, we discovered that the financial implications of the decision to be made had an impact on whether or not women had a say in making that decision. We investigated the decision to buy a large item such as a refrigerator, the decision to purchase a productive asset such as land and the decision to build a house. We found that for these three decisions, between 15 and 25% of the women left the decision making to their partners. Propensity to do so was linked to age; the youngest women were less likely to leave the decision making to their partners than the oldest women. For example, while only 11.6% of the youngest women and 12% of the middle aged women left the decision to build a house to their partners to make, the same was true for twice as many women aged above 50 (23%). Although, with each of these three decisions, the percentage point difference between the youngest and oldest group was more than 10, none of these differences was statistically significant reflecting yet again a non-significant change in decision making abilities over the two generations (*p* = 0.258 for large purchases; *p* = 0.324 for purchase of a productive asset and *p* = 0.242 for building a house; see Table 1).

**Table 1:** **Table showing decision making and related preferences of Ghanaian women across three generations**.

	**Age**			
	**18–29**	**30–49**	**50+**	**Total**
Percentage of women who would keep a baby even if partner does not want it	19.8	16.7	13.9	16.8
Percentage who had a say in who they married	92.9	88.3	79.3	86.8
Percentage who believe that women should derive pleasure from sex	91.3	93.1	85.4	90.0
Percentage who have ever used a contraceptive	42.7	53.0	36.7	44.1
Percentage who left the decision to build a house to their partner	11.6	12	23.0	15.2
Percentage who left the decision to buy a productive asset to their partner	10.8	18.2	25.0	18
Percentage who left the decision to buy a large item to their partner	20.4	26.0	29.0	25

Source: Everyday Life Survey, 2008

Decision making in the area of reproduction differed markedly from other areas. While the majority of women reported believing that women should derive pleasure from sex (average of 90%), (in stark contrast to conceptualizing sex as being solely for procreation), contraceptive use (previous and current) was common in less than half of the sample, with the lowest rate in the 50+ age group. A domain in which women of all age groups sampled would largely defer to their partner's position (at least 80% of the time) was in a scenario where the woman was pregnant but their partner did not want the baby. Women stated that in such situations they would keep the baby on average 16.8% of the time. Women 50 years and older were the least likely to report that they would keep the pregnancy despite their partner's wishes. Basically, women, while believing that sex was not strictly for procreation were doing little to ensure that sexual activity did not result in procreation. When it did, the ultimate decision as to what to do was also left largely to their partners.

Our results in decision making are consistent with extant research on gender in Ghana. According to the national census, only 0.2% of Ghanaian women serve in managerial and administrative positions. Amu (2005) observes that this low level of female representation at the top of organizational hierarchies results in the exclusion of women from decision-making at the macro-institutional level. Thus, while traditional values once kept women from making decisions that affected their own lives, and despite the fact that women are becoming more autonomous in making important decisions that affect their households (except in the area of reproduction), inequalities in access to higher education largely constrain Ghanaian women's access to jobs that put them in positions to make decisions that impact the larger society.

There is marked similarity between trends in the 2010 national sample and that of the 2008 Everyday Lives survey, suggesting that at the national level not much has changed in the lives of Ghanaian women over this 2 year period. This assertion is consistent with data from The Global Gender Gap Index (Hausmann et al., 2013) which measures and ranks gender-based access to resources and opportunities within individual countries, specifically in the areas of economic participation, educational attainment, health and survival, and political empowerment. Ghana's Gender Gap Index score did not change by much between 2006 and 2013, due to a predominant stabilization of index scores over this 7 year period.

## Psychological implications and conclusions

*The younger generation say: “We no longer rest in the ancient resting place.” Why then do they not throw away one of the three old hearthstones and leave two? (Bu Me Be #1766). (Used in warning people against discarding all old traditions as useless. They are so much a part of man that he cannot do without them. Three hearth stones are used to balance the cooking pot)*.

On the one hand, Ghanaian society is changing. As demographic trends reviewed in the introduction indicate, Ghanaian women are more involved in spousal selection (Takyi, 2003), are more likely to head households (Ardayfio-Schandorf, 2006), and have fewer children (Ghana Statistical Service, 2008) than they did in previous times. Such changes are expected, given the impact of modernization on Ghanaian society, and deliberate efforts at a national level to improve the status of women (e.g., in terms of access to land, education, legal services, and microcredit). The assertion that individuals are active shapers of the culture that shapes them (Markus and Kitayama, 2010) brings to mind the notion that women are active in the determination of how womanhood is and will be interpreted and defined. As women embrace, reject, maintain, and champion changes in today's Ghanaian society, these behaviors impact how being a woman is perceived.

On the other hand, as illustrated by the proverb above, Ghanaian women have not discarded their hearthstones. Ghanaian culture promotes an interdependent way of being in the world (Adams and Dzokoto, 2003; Adams, 2005). The cultural foundation of relational interconnectedness actively influences many aspects of the lives of Ghanaian women, some of which have been examined in this paper. Our data suggests mostly a lack of statistically significant differences between different age groups on issues concerning autonomy and decision-making in the lives of Ghanaian women. This implies maintenance of the cultural norm of agency within an interconnected social space: many decisions made by women are accomplished with the involvement of others.

What sustains this cultural norm in the face of the aforementioned social changes? What encourages women not to discard the hearthstone? A possible factor could be that particularly in collectivistic societies, cultural fit, the degree of congruence between an individual's values and those prevalent in their cultural environment, is important for individual wellbeing (see Kitayama et al., 2000; Hovey et al., 2006; Hamamura et al., 2009; Li and Hamamura, 2010). As explored through proverbs, the role of the “good/ideal Ghanaian woman” involves significant contributions to the nuclear family (e.g., in terms of economic support, and bearing and raising children), and an amenability to influence and support from the extended kinship network. Shifts away from such an interdependent construction of the self—particularly without the support of an alternate supportive cultural institution (such as a charismatic church; see Salter and Adams, 2012)—may have negative consequences for an individual's wellbeing. For instance, a woman with two children—who is personally happy with the number of children she has—may succumb to familial pressure (through repeated nagging, and advice from multiple kin) to have one more though she may not necessarily perceive it financially prudent to do so. The goal of preservation of social harmony may make it culturally adaptive not to discard the “traditional hearthstone” in a modern world.

It would be remiss not to acknowledge that our study has several limitations. As previously discussed, it is impossible to tie the values espoused by proverbs to specific historical periods. Concurrence with proverbial messages may have changed over time. Furthermore, proverbs communicate the collective norm (culture outside the self) and do not address individual differences. Also, while our findings were consistent with extant literature on gender in Ghana, our quantitative research design—cross-sectional data collected at one point in time—does not allow for the exclusion of the possibility of the influence of age and cohort effects in the survey data. Future research should address these constraints.

### Conflict of interest statement

The authors declare that the research was conducted in the absence of any commercial or financial relationships that could be construed as a potential conflict of interest.
