# Supplementary material for: If times change, should we throw away the hearthstone? Exploring (Dis) continuities in autonomy and decision-making in the lives of Ghanaian women
Source: Front Psychol. 2014 Nov 28;5:1371. doi: 10.3389/fpsyg.2014.01371 (PMC4246668; doi:10.3389/fpsyg.2014.01371)
Supplement: Supplementary file 1 [file DataSheet1.DOCX]

Appendix A: Extract from the 2008 Everyday Life Survey

1. If you are pregnant and want the baby, can you make the final decision too have the baby even if your partner says no?

a) Yes b) No

1. Did you have a say in who you entered into a marriage with the first time?

a) Yes b) No

1. Have you used any contraceptive method before?

a) Yes b) No

1. Do you believe that you should derive pleasure from sex?
2. Yes b) No
3. In your household, who makes the decision to build a house?

a) I am the major decision maker

b) My husband is the major decision maker

c) Both my husband and I make this decision

d) Other family members make the decision

e) Non-kin make the decision

f) Not applicable

1. In your household, who makes the decision to purchase a productive asset?

a) I am the major decision maker

b) My husband is the major decision maker

c) Both my husband and I make this decision

d) Other family members make the decision

e) Non-kin make the decision

f) Not applicable

1. In your household, who makes the decision to buy a large item?

a) I am the major decision maker

b) My husband is the major decision maker

c) Both my husband and I make this decision

d) Other family members make the decision

e) Non-kin make the decision

f) Not applicable
